# The Gut Microbiome: a New Frontier in Musculoskeletal Research

**DOI:** 10.1007/s11914-021-00675-x

**Published:** 2021-04-17

**Authors:** R. Li, C. G. Boer, L. Oei, Carolina Medina-Gomez

**Affiliations:** 1 Department of Internal Medicine, Erasmus MC University Medical Center, Rotterdam, The Netherlands; 2 Department of Epidemiology, Erasmus MC University Medical Center, Rotterdam, The Netherlands; 3 Department of Internal Medicine, Leiden University Medical Center, Leiden, The Netherlands

**Keywords:** Gut microbiome, Musculoskeletal system, Sarcopenia, Osteoporosis, Osteoarthritis, Rheumatoid arthritis

## Abstract

**Purpose of the review:**

The human gut harbors a complex community of microbes that influence many processes regulating musculoskeletal development and homeostasis. This review gives an update on the current knowledge surrounding the impact of the gut microbiota on musculoskeletal health, with an emphasis on research conducted over the last three years.

**Recent findings:**

The gut microbiota and their metabolites are associated with sarcopenia, osteoporosis, osteoarthritis, and rheumatoid arthritis. The field is moving fast from describing simple correlations to pursue establishing causation through clinical trials.

**Summary:**

The gut microbiota and their microbial-synthesized metabolites hold promise for offering new potential alternatives for the prevention and treatment of musculoskeletal diseases given its malleability and response to environmental stimuli.

## Introduction

The musculoskeletal system is an important determinant of overall human health. Besides serving as a scaffold for the body and its locomotive function, it is in constant communication with other organs in the body through biochemical signaling, increasingly recognized to harbor fundamental endocrine functions. For instance, it has been postulated that bone can exert critical functions regulating male fertility and whole-body glucose metabolism [1] and that myokines (i.e., cytokines and other peptides released by muscle fibers) can influence cognition, lipid and glucose metabolism, browning of white fat, bone formation, endothelial cell function, muscle hypertrophy, skin structure, and tumor growth [2].

Musculoskeletal disorders constitute a major cause of disability and morbidity [3]. The economic burden of these diseases on health systems worldwide is predicted to continue increasing together with life expectancy. This global challenge requires urgent and feasible solutions. The pharmacological treatment of most skeletal conditions is broad, ranging from anti-inflammatories and analgesics to topical preparations and nutraceuticals [4], whereas no pharmacological treatment exists for instance for sarcopenia. There is also a lot of emphasis on lifestyle-modification approaches, including physical activity and diet changes to improve the prevention and treatment of musculoskeletal diseases.

Among the novel approximations to preserve musculoskeletal health, the study of the gut microbiota (GM) and their microbial-synthesized metabolites holds promise offering new potential alternatives for the prevention and treatment of musculoskeletal diseases, as diet and lifestyle modifications can impact the composition, richness, and predicted functional profiles of the gut microbiota [5]. The GM encompasses a set of over 2000 different kinds of microorganisms residing in our gastrointestinal tract, encoding 150-fold more genes than the human genome [6]. These microbial communities are assembled during the first 2 years of life after which they stabilize; as such, disruption of this colonization at early ages could affect maturation and developmental pathways [7]. Unsurprisingly, microbiome metabolites are now believed to influence numerous diseases, such as cancer, diabetes, cardiovascular disease, multiple sclerosis and autism spectrum disorder, amid many others [8]. Besides this, the GM strongly interacts with certain drugs and influences their action [9]. The GM is now considered to be the leading edge of scientific research accounting for more than 9500 research publications in the last year [9], and the musculoskeletal field is no exception. The last year has witnessed an upsurge in research on the viral component of the microbiome (i.e., the virome), which is dominated by bacteriophages, determinant in shaping bacterial communities [10]. Their study is essential to bridge gaps of knowledge on the ecology and functionality of the GM.

There is an increasing body of evidence showing that the GM can exert effects in the musculoskeletal system as it modulates gut permeability, hormonal secretion, and immune response, and stimulates calcium and vitamin D absorption [11]. Therefore, the modulation of the GM could be seen as a next-generation treatment for musculoskeletal disorders. The influence of the gut microbiome on musculoskeletal health and disease processes can be direct or indirect. For example, the GM can play key roles in the success of lifestyle interventions aimed at mitigating the impact of aging in the musculoskeletal system or the development of disease. In this review, we will provide an overview of studies that examined the impact of the GM or their products on the musculoskeletal system, with special emphasis on the effects targeting bone homeostasis through the life course.

## Gut Microbiome as Determinant of Musculoskeletal Health and Disease

In this review, we prioritized work published during the last three years, strategy that may have resulted in underrepresentation of previously published high-quality work and reviews on this topic [11–19]. We cover in detail the relationship with skeletal outcomes i.e., bone metabolism/osteoporosis, but also provide an overview of its significance to muscle function/sarcopenia, cartilage integrity/osteoarthritis and its role on the immune response/specifically in rheumatoid arthritis.

## Gut Microbiome Effects on Bone Metabolism

Bone metabolism depends on the balance between bone formation and resorption orchestrated by the action of osteoblasts, osteocytes, and osteoclasts. Bone mass starts to be accrued after birth and peaks in young adulthood, decreasing thereafter [20]. A high peak bone mass is associated with reduced osteoporosis risk in later life, with simulation studies showing that a 10% increase in peak bone mass delays the onset of osteoporosis by 13 years [21]. Therefore, measures intending to maximize peak bone mass and strength are important when designing strategies aimed at reducing the risk of osteoporosis or low bone mass later in life. Osteoporosis is a disease affecting > 200 million elderly worldwide, characterized by increased microstructural deterioration of bone tissue and low bone mass which ultimately leads to fragility fractures. Despite a range of effective compounds to reduce fracture risk, treatment rates are low among high-risk individuals [22].

**Fig. 1:**
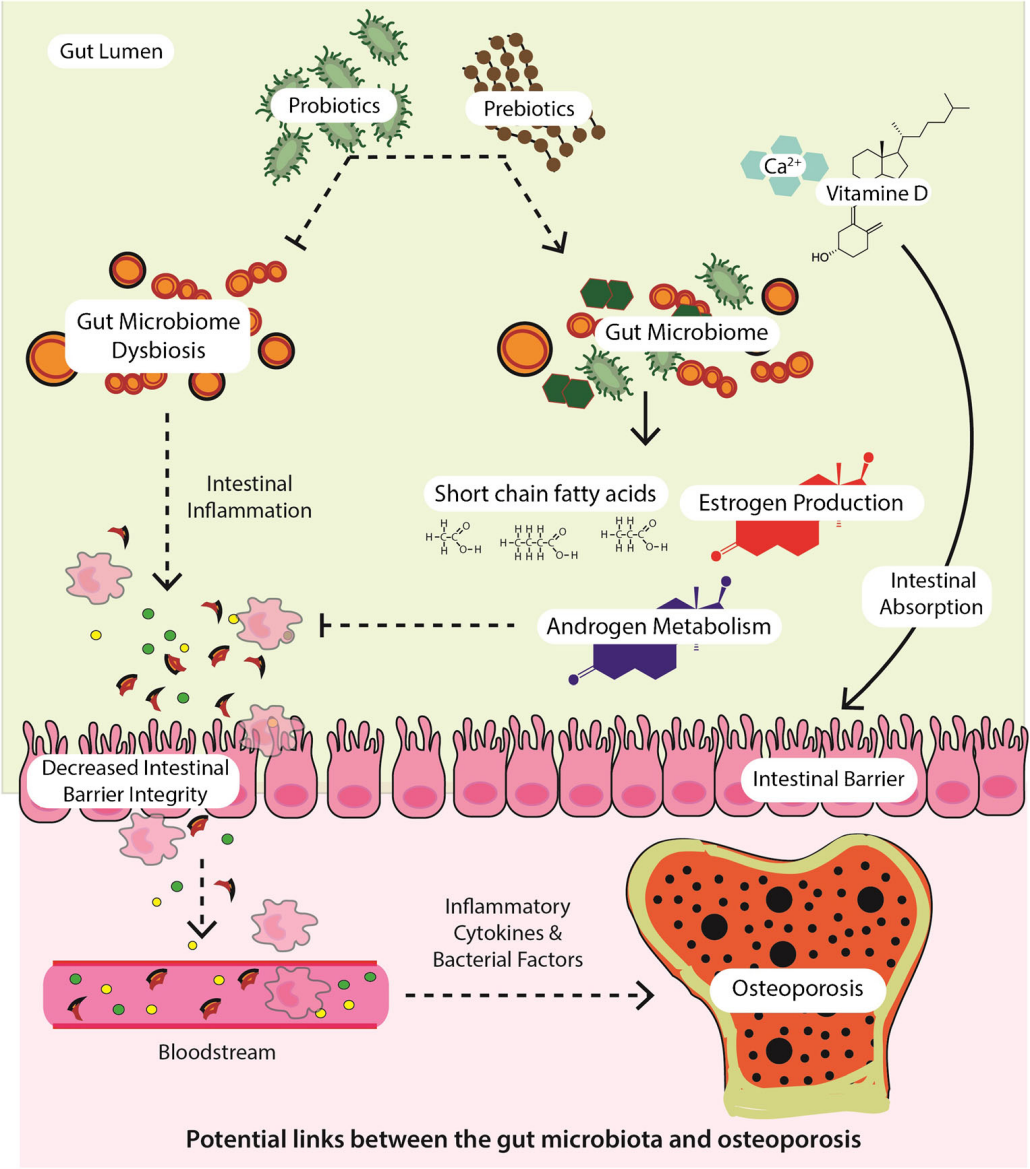

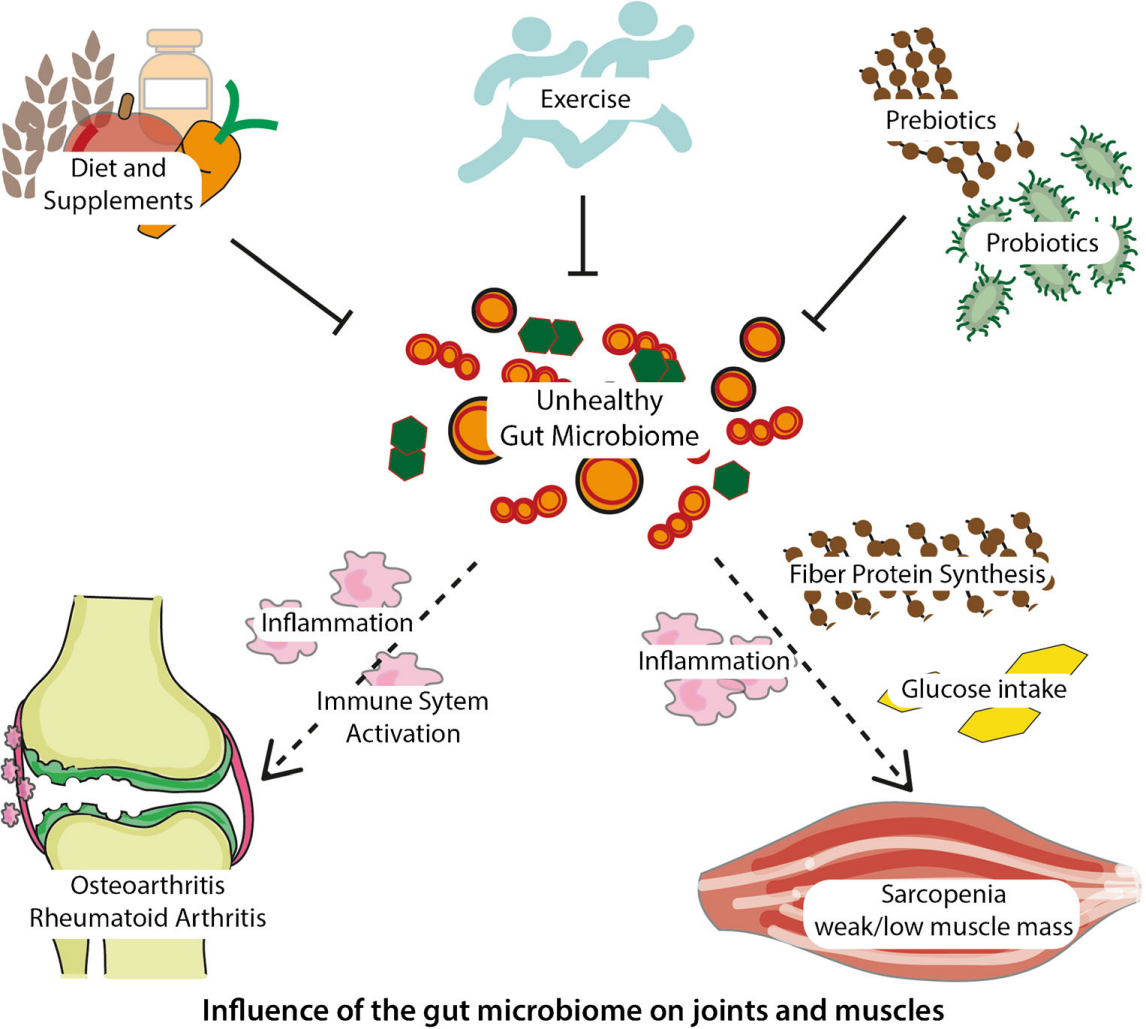
**a** Influence of gut microbiota on bone. The gut microbiota (GM) contributes to preserve gut barrier integrity. The GM affects absorption of calcium and vitamin D, maturation of the immune system, and production and activation of hormones as estrogens and androgens. Moreover, GM dysbiosis can result in production of inflammatory cytokines that translocate intestinal barrier and exert detrimental effects on bone. Probiotics and prebiotics have shown potential to mitigate or restore bone health. **b** Influence of gut microbiota on joints and muscles. The GM can influence joint health through some mechanisms including host immunity, and inflammation. Whereas influence of muscle health involves also glucose intake, energy metabolism, and fiber protein synthesis

With over 435 publications appearing in PubMed between the years 2013 and 2019, microbiome research applied to bone health is clearly booming. This is not unexpected, considering that host metabolic pathways, the immune system, and the hormonal environment constitute important determinants of bone metabolism. Consequently, it is rational to think that the GM also plays an important role in bone homeostasis (Fig. 1). Indeed, the risk of osteoporosis has been associated with inter-individual variation of the gut microbiome [23]. Understanding relevant host-microbe interactions would in principle open the door to better diagnostic and therapeutic options in osteoporosis management. Here, we summarize research illustrating different routes by which the GM affects bone, derived mainly from work in animal models and evidence emerging from human studies. This work has evolved from describing simple correlations to pursue establishing causation through clinical trials.

### Nutrition and Bone Development

Calcium and vitamin D are key bone health nutrients, whose depletion or deficit results in adverse skeletal complications [24]. These nutrients have been considered critical, to the point that clinical trials aimed at showing the effectiveness of osteoporosis medications systematically include vitamin D and calcium as part of the treatment regimen [25–27]. Yet, over the last years, a growing number of studies have questioned the use of supplementing these nutrients in the general population [28–30], in contrast to their established benefit in deficient individuals. During skeletal development, calcium and vitamin D exert critical roles [31] and the involvement of the GM in the absorption and activation of these nutrients might then be of great significance during early ages. Another molecule to consider is vitamin K, which albeit some contradictory findings has shown a positive effect on bone health [32].

#### Vitamin D

Childhood vitamin D deficiency is considered a significant public health issue around the world [33]. Randomized controlled trials of vitamin D supplementation in children with deficiency have shown improvement on bone mineral density (BMD) [34]. Besides its direct effect on calcium absorption, vitamin D regulates the homeostasis of the gut mucosa by maintaining the integrity of the epithelial barrier and thus the translocation of microbial metabolites to the host. This regulation also influences the maturation of the immune system and inflammation responses [35]. With respect to its relation with the GM, a recent small intervention study showed that high vitamin D supplementation in adolescent girls (i.e., 9 weekly doses of 50,000 IU) resulted in an increase of *Firmicutes*, *Bifidobacterium*, and *Enterococcus* and a decrease of *Bacteroidetes* and *Lactobacillus* [36]. In line with these findings, vitamin D receptor (VDR) knock-out (KO) mice have a microbiome enriched for *Bacteroides* and *Clostridium* but depleted in *Lactobacillus* [37]. In addition, these mice had alterations in metabolites specifically produced from carbohydrate, protein, lipid, and bile acid metabolism [38]. These studies support the contention that vitamin D regulates the GM, also supported by the identification of variants in the VDR gene in a GM genome-wide association study (GWAS) [39]. Conversely, there is also evidence of the GM influencing the levels of circulating vitamin D. For instance, a clinical trial in 127 individuals showed that supplementation of *Lactobacillus reuteri NCIMB 30242* increased mean circulating 25-hydroxyvitamin D by 25.5% after a 9-weeks intervention [40]. On top of this, bacteria as *Streptomyces griseolus* can hydroxilize vitamin D3, essential step to its activation, as human metabolic enzymes do [41].

#### Calcium Absorption

Calcium is the most common mineral in the human body. For a high peak bone mass to be achieved, the intake of calcium needs to be adequate, particularly during periods of rapid growth when absorbed calcium is retained, rather than excreted in the urine [42]. Several studies have demonstrated that short-chain fatty acids (SCFAs) produced by the GM help improving calcium absorption in humans and increase bone density and strength in animal models [43–45]. These studies focused on the advantage of consumption of dietary fibers and their effect in the increment of SCFAs as well as *Parabacteroides*, *Bifidobacterium*, and *Bacteroides* relative abundances (a nice review on the topic can be found [46]). The importance of these studies lies in the use of prebiotics (i.e., compounds that induce the growth or activity of beneficial microorganisms) or postbiotics (i.e., factors secreted by live microorganisms) to correct calcium deficiency without the need for an increase in calcium-rich foods or supplements. However, there are studies suggesting that the relationship between the GM and calcium levels is not unidirectional. Calcium supplementation has been shown to increase the microbial diversity and the number of *Bifidobacterium sp* [47], *Ruminococcaceae*, and *Akkermansia* in mice [48]. Also, in healthy men, intake of 1000 mg of calcium and 1000 mg of phosphorus per day, for 8 weeks, increased the fraction of *Clostridium XVIII* in the fecal samples [49].

#### Vitamin K

It has been shown that vitamin K is implicated in bone health by promoting the osteoblast-to-osteocyte transition, limiting osteoclastogenesis and intermediating the process of osteocalcin carboxylation [32]. However, a recent study has also implicated this vitamin in changes in the composition and structure of the organic or mineral material [50]. By using metagenomic analysis of the fecal microbiota from mice as well as nanoscale chemical analysis of bone tissue, the authors were able to identify reductions in the concentrations of forms of vitamin K generated by microbes in mice with impaired bone strength [50].

#### Dietary Fibers

Despite the known caveats of nutrition epidemiology such as compositionality of the data and correlation with varying social and behavioral factors [51], two large epidemiological studies have shown a positive effect of fiber intake on bone outcomes [52, 53]. It is hypothesized that the effect of fibers in health is at least partially explained by their fermentation to SCFAs (acetate, propionate, butyrate) by the GM. Besides the positive effects of SCFAs already described, administration of propionate (C3) or butyrate (C4) prevents ovariectomy-induced, as well as, inflammation-dependent bone loss in mice [44]. Butyrate can also inhibit histone deacetylase and stimulate osteoblast differentiation [54] and increase bone formation with increased bone sialoprotein and osteoprotegerin production [55]. Moreover, butyrate could stimulate bone formation via T regulatory cell-mediated regulation of *WNT10B* expression [56]. Mechanistically, C3 and C4 induce metabolic reprogramming of osteoclasts, resulting in enhanced glycolysis at the expense of oxidative phosphorylation, thereby downregulating essential osteoclast genes such as *TRAF6* and *NFATc1* [44]. Reduction of osteoclast differentiation was also recently observed in the alveolar bone in mice in response to SCFAs administration [57]. In line with these results, mice fed with a diet rich in short-chain Galacto-Oligosaccharides and long-chain Fructo-Oligosaccharides (scGOS/lcFOS), prebiotics used by GM as substrate for the production of SCFAs, showed an improved BMD [58].

### The Role of Hormones on Bone Metabolism

#### Insulin-Like Growth Factor 1 (IGF-1)

IGF-1 plays an essential role in regulating skeletal development and postnatal growth. *Igf1* KO mice exhibit decreased post-natal growth rate and delayed skeletal ossification, whereas overexpression of *Igf1* significantly increases radial bone growth in male and female mice [59, 60]. IGF-1 serum levels have been shown to be higher in mice with an intact GM as compared with germ-free (GF) mice. As expected, GF mice showed decreased linear growth, femur length, cortical thickness, and trabecular bone [59]. Administration of SCFAs was sufficient to increase circulating IGF-1 [59]. However, a study performed in a different mice strain showed the opposite results [61]. These seemingly contradictory findings might be explained by the specific genetic background of the experimental subjects and/or age-dependent effects. Viruses of the *Irdoviradae* family in the human gut virome have been shown to produce viral insulin/IGF-1-like peptides (VILPs). These peptides are able to bind to murine and human IGF-1 receptors and stimulate cell growth in vitro [62].

#### Sex Hormones

The estrogen depletion observed in post-menopausal women adversely impacts bone homeostasis, and one of the principal regulators of circulating estrogens is the GM [63]. The GM regulates estrogens through the secretion of β-glucuronidase, an enzyme that deconjugates estrogens into their active forms. When this process is impaired through, for example, lower diversity of the GM, the decrease in deconjugation results in a reduction of circulating estrogens [63]. Excessive osteoclast formation and resorption are considered as the key pathological changes in estrogen-deficiency-induced osteoporosis [64]. Moreover, estrogen deprivation increases intestinal permeability allowing the translocation of bacteria and increasing the number of antigens entering the epithelial mucosa what could lead to systemic inflammation. Compared with normal mice, GF mice showed less bone loss, following estrogen deficiency, due to the reduction of osteoclastogenic cytokines [59]. In addition, probiotic treatment based on different *Lactobacillus* species reduced the expression of osteoclastogenic cytokines and increased the expression of OPG in bone, protecting mice from ovariectomy (OVX)-induced bone loss [65]. *Bifidobacterium longum* has also been reported to alleviate bone loss in OVX rats [66]. Further, treatments to prevent gut leakage either by antibiotic depletion of the gut microbiota or administration of *Lactobacillus reuteri* were shown to be effective in the treatment of glucocorticoid-induced osteoporosis in mice [67]. Androgens, other type of sex hormones, are also essential for bone development and maintenance [68]. Recently, it has been shown that the GM modulates levels of free hydrotestosterone (DHT), a potent androgen, in the distal intestine. However, further studies are necessary to clarify if the GM has the capacity to regulate androgen metabolism and action, also at extra intestinal locations [68].

### Role of the Microbiota in Immunity and inflammation

The GM plays a central role in the maturation of the immune system. It is involved in the production of circulating cytokines and the development of lymphoid cells, particularly of T-helper lymphocytes. With age and particularly in response to estrogen deficiency, T cells increase their production of pro-inflammatory and pro-osteoclastogenic cytokines, such as TNF-α and RANKL [69]. The ability of the GM to increase these cytokines and reduce cortical bone in mice is actually dependent on NOD1 and NOD2 signaling which elicits an inflammatory response [70]. Studies have also shown that activation of the toll-like receptor 5 (TLR5), another pattern recognition receptor used by the innate immune system, prompts osteoclast formation and bone loss in mice. Besides, TLR5-KO mice present with increasing periosteal expansion [71], which is normalized when there is a disruption of the GM, in line with a mediation role of the GM.

Exercise is another integral component of osteoporosis management, as physical activity increases BMD and reduces inflammatory markers [72]. Recently, it was proposed that exercise may prevent bone loss through changes in GM. This was based on the results of an activity study in mice, where members of the *Bifidobacteriaceae* family, known to reduce intestinal inflammation, positively correlated with BMD [73].

## Clinical Studies Assessing the Effect of GM in Osteoporosis

### Observational Studies

An association study by Das *et al*. found a higher abundance of *Actinomyces*, *Eggerthella*, *Clostridium Cluster XlVa*, and *Lactobacillus* genera in individuals with osteoporosis (*N* = 60) compared with individuals with normal BMD (*N* = 60) and a lower abundance of *Escherichia/Shigella* and *Veillonella* species in the osteoporotic individuals compared with an osteopenic group (*N* = 61) [74]. No statistical differences were found in diversity metrics among the groups [74]. In contrast, another study found higher diversity and a higher abundance of *Dialister* and *Faecalibacterium* in individuals with osteoporosis (*N* = 48) as compared with individuals with normal levels of BMD (*N* = 48) [75], whereas two Chinese studies, each in about hundred individuals, reported correlations between the abundance of *Bifidobacterium, Roseburia*, and *Lactobacillus* [76] and *Allisonella, Klebsiella*, and *Megasphaera* [77] and BMD, respectively. These inconsistent results show the importance of using adequate sample sizes and controlling for multiple testing when investigating possible new associations.

### Microbiome-Based Clinical Trials

Two different clinical trials carried out in Sweden have shown a substantial decrease in bone loss in postmenopausal women after probiotic use. The first one, enrolled 90 postmenopausal women and showed that after 1 year of daily supplementation with *Lactobacillus reuteri 6475*, the treatment group presented reduced volumetric BMD loss at the tibia (mean difference between groups =1.02%; 95% CI: 0.02–2.03%) [78]. The second one focused on bone loss at the lumbar spine (LS). Two hundred thirty-two early postmenopausal women completed the trial in which half of them (116) received probiotic treatment consisting of a daily dose of three *Lactobacillus* strains (*Lactobacillus paracasei DSM 13434, Lactobacillus plantarum DSM 15312, and Lactobacillus plantarum DSM 15313*; 1×10^-10^ colony-forming units per capsule) per 12 months or placebo. LS-DXA scans were taken the day of intake and 1 year later when the treatment ended. *Lactobacillus* treatment reduced the LS-BMD loss compared with placebo (mean difference 0.71%, 95% CI 0.06 to 1.35) [79]. The LS-BMD loss was significant in the placebo group (–0.72%, −1.22 to −0.22), whereas no bone loss was observed in the *Lactobacillus*-treated group (–0.01%, −0.50 to 0.48) [79]. The authors concluded that the *Lactobacillus* strains seem to target mechanisms with differential action on trabecular and cortical bone. Conversely, a clinical trial in 76 healthy postmenopausal Japanese women observed a positive effect of administration of *Bacillus subtilis C-3102* for 24 weeks in total hip BMD (placebo = 0.83 ± 0.63%, C-3102 = 2.53 ± 0.52%, *p* =0.043), whilst no significant effect was observed in the LS-BMD of the participants taking the probiotic as compared with the placebo group. Based on microbiome profiles, urinary type I collagen cross-linked N-telopeptide and tartrate-resistant acid phosphatase isoform 5b measurements the authors presume that *C-3102* improves BMD by inhibiting bone resorption and modulating the GM [80]. In a study comprising 50 healthy post-menopausal Iranian women [81], a lower serum collagen type 1 cross-linked C-telopeptide (CTX) was also detected in the intervention group as compared with the placebo group. The intervention group, comprising 25 women, took a multispecies probiotic capsule (GeriLact) daily for 6 months. GeriLact contains *Lactobacillus casei, Bifidobacterium longum, Lactobacillus acidophilus, Lactobacillus rhamnosus, Lactobacillus bulgaricus, Bifidobacterium breve*, and *Streptococcus thermophilus* [81]. The presence of reduced bone turnover was also supported by lower levels of bone-specific alkaline phosphatase (BALP) in the intervention group after treatment [81].

### Gut Microbiome Effects on Skeletal Muscle Mass and Function

Even if the gut-muscle axis has not been studied to the extent of the gut-bone axis, this field is gaining momentum [82–88]. This axis may be involved in the pathogenesis of muscle wasting disorders through the transduction of pro-anabolic stimuli from dietary nutrients, modulation of inflammation and insulin sensitivity among others (Fig. 1) [19]. It has been shown that skeletal muscle mass and physical function are reduced in GF and in antibiotic-treated mice [83, 85, 89]. Transplanting the GM from conventionally raised mice to GF mice resulted in an increase in skeletal muscle mass and a reduction in muscle atrophy markers [85]. Moreover, merely treatment with SCFAs partly reversed skeletal muscle impairments in these mice [85]. Another study demonstrated that the reduced physical fitness, exercise performance, and energy metabolism in young GF mice could be improved by inoculation of either *Eubacterium rectale, Lactobacillus plantarum TWK10*, or *Clostridium coccoides* [87]. Similarly, reduced running endurance in conjunction with increased ex vivo muscle fatigability was found in antibiotic-treated mice [83], which could be entirely normalized by natural reseeding of the gut microbiota [83]. In humans, genus *Prevotella* and *Barnesiella* have been shown to be more abundant in the fecal samples of elderly with higher lean mass and better physical performance as compared with low-functioning elderly in a small study (high-functioning, *N* = 18; low-functioning, *N* = 11) [88]. Colonization of GF mice with the human microbiota of the highly functional individuals resulted in higher grip-strength in these mice. However, no differences were observed in total lean mass or endurance between mice colonization with the high-functioning human microbiota or low-functioning microbiota [88]. Currently, there is an ongoing clinical trial in Ireland aiming to assess the effect of *Bacillus coagulans* as probiotic on the rates of muscle protein synthesis [90]. If *Bacillus coagulans* supplementation can improve muscle protein synthesis rates following plant protein consumption, then that could embody an effective and environmentally sensitive strategy to attenuate adverse age-related loss of muscle mass and physical function in the elderly.

### Gut Microbiome Effects on the Joints

Osteoarthritis (OA) is the most common chronic degenerative joint disease and a leading cause for joint disability worldwide [91]. Currently, no curative treatment exists for OA. Well-established risk factors for OA are obesity and macrophage-mediated inflammation, both linked to the GM (Fig. 1) [92, 93]. In principle, different mechanisms by which the GM can reduce obesity would have a high impact on modifying OA risk. Accordingly, mice following a long-term high-fat diet are prone to develop obesity-mediated OA. However, this risk is reduced by intervention with *Lactobacillus paracasei* subsp. *paracasei M5* or the prebiotic oligofructose [94]. In addition, a small-scale study recently demonstrated worsening of OA pathology in the presence of serum and synovial fluid containing high bacterial LPS (lipopolysaccharides) levels with activated macrophages in the knee joint capsule and synovium [95]. Moreover, the abundance of *Streptococcus* species was recently associated with increased knee pain and knee joint inflammation in a large population study of older adults [96]. Clinical trials in humans have already shown a positive effect of *Lactobacillus casei Shirota* [97] and *Streptococcus thermophilus* [98] in the progression of knee OA.

### Gut Microbiome Effects on the Immune Response

Rheumatoid arthritis (RA) is an autoimmune disease in which systemic chronic inflammation leads to joint destruction. Altered composition of the oral and gut microbiota has been observed in RA both in mice and human studies (reviewed recently in [17]). Several GM bacteria species have been found to be enriched in RA cases, among which *Prevotella* species [99] and different species of *Lactobacillus*. Also, the oral microbiome species *Cryptobacterium curtum* has been found to be enriched in RA cases. This bacterium is capable of producing large amounts of citrulline which is known for acting as an autoantigen in RA [100]. Also in relation to RA, *Lactobacillus casei* was able to suppress the induction of RA and protect bones from destruction in a study using rats [101].

## Future Perspectives

It is clear that the GM presents an exciting new frontier in musculoskeletal research. Although the potential benefit of GM research is high, several hurdles still need to be overcome before GM research can be translated to the clinic. The majority of the GM research related to (musculoskeletal) health has been done using 16S ribosomal RNA (rRNA) sequencing. The 16S rRNA technique, while technically robust, has limited resolution to identify specific bacteria related to disease (i.e., taxonomical orders bellow genus level) or to assess functional potential, as opposed to for example shotgun metagenomics sequencing. This limitation hampers the possibility of translating microbiome research to the development of advanced therapeutics. Moreover, most microbiome studies have been carried out in small sample sizes that are not representative of the population and can be hindered by the high inter-individual variability of the GM and high dimensionality of the data. On top of this, methods and procedures for collection, extraction, and analysis of microbiome data are not standardized across studies, which has led to a lack of reproducibility in the field. Therefore, harmonization of large-scale studies will allow guarantying reproducible science. As large GWAS of GM variability continue to emerge, leveraging genetic information to construct instrumental variables allowing systematic exploration of unconfounded relationships between GM and musculoskeletal traits starts to materialize using the Mendelian randomization approach [102]. Recently, the relationship of a microbiome-based polygenic risk score and BMD was assessed in the UK Biobank and found only one nominal significant association with pelvic BMD [103]. However, the GM-GWAS selected by the authors as the base for their analyses were rather small [103]. The recent publication of the MiBioGen consortium meta-analysis including more than 18,000 individuals [104] opens the opportunity to evaluate the association of multiple microbes and musculoskeletal health outcomes in well-powered settings. This strategy, will also allow the efficient designs of clinical trials, with increased likelihood of success. Altogether, the efficacious clinical trials summarized here are likely to fuel further studies and the development of new therapies based on our ever-growing knowledge of the relation of GM and bone metabolism. Moreover, patient surveys in the USA have shown the great acceptance of prebiotics and probiotics suggesting that microbiome-based therapies could increase treatment compliance in patients [105, 106].

Last but not least, the characterization of the gut virome is still in its infancy. Yet, viruses can alter microbiota structure by infecting specific populations of bacteria as well as trigger apoptosis or induce alterations in host cells [107]. Therefore, they are intrinsically involved in the maturation of the immune system and the inflammation process. Given these findings, it is timely to evaluate the potential role of the gut virome in musculoskeletal health and disease.

## Concluding Remarks

This review highlights studies in which the GM is shown to have not just an association but also a key modulatory role in the musculoskeletal system. There is substantial space for improving the current management of musculoskeletal diseases, and the GM-derived treatments are an exciting point of inflection with growing number of opportunities. This increasing body of evidence, together with the GM response to environmental stimuli and malleability, positions it as a potential crucial driver of the personalized healthcare revolution, including musculoskeletal diseases.
